# Profiling the Complexity of Resistance Factors in Cancer Cells Towards Berberine and Its Derivatives

**DOI:** 10.3390/ph19010027

**Published:** 2025-12-22

**Authors:** Nadire Özenver, Nadeen T. Ali, Rümeysa Yücer, Xiao Lei, Gerhard Bringmann, Thomas Efferth, Mona Dawood

**Affiliations:** 1 Department of Pharmacognosy, Faculty of Pharmacy, Hacettepe University, 06100 Ankara, Türkiye; nadire@hacettepe.edu.tr; 2 Department of Pharmaceutical Biology, Institute of Pharmaceutical and Biomedical Sciences, Johannes Gutenberg University, 55128 Mainz, Germanyefferth@uni-mainz.de (T.E.); 3 Institute of Organic Chemistry, University of Würzburg, Am Hubland, 97074 Würzburg, Germany; gerhard.bringmann@uni-wuerzburg.de

**Keywords:** alkaloids, cancer, cluster analysis, drug resistance, live cell microscopy, phytochemicals, proteomics

## Abstract

**Background:** Berberine, a benzylisoquinoline alkaloid, has been traditionally used in Ayurvedic and Chinese medicine. We examined the resistance mechanisms to berberine in a panel of different cancer cells and focused on understanding its molecular mechanisms. **Methods:** Resazurin assay determined berberine’s cytotoxicity. Molecular docking unraveled the interaction of berberine with the BCRP transporter. Fluorescence microscopy evaluated its effect on microtubules. Further, proteomic profiling identified novel determinants of cellular response to berberine and its derivatives. **Results:** Cell lines overexpressing ABC transporters displayed cross-resistance to berberine compared to their counterparts. While cells over-expressing EGFR were 3.57-fold resistant, wild-type and p53 knockout cells showed similar sensitivity to berberine. P-glycoprotein/*ABCB1*, *EGFR*, and *WT1* expression correlated with the log_10_IC_50_ values for berberine in the NCI cell line panel. Berberine was bound to the same pharmacophore of BCRP as BWQ, and live cell microscopy showed that *BCRP*-transfected cells did not uptake considerable amounts of berberine in contrast to wild-type cells. Berberine altered the microtubule cytoskeleton similarly to vincristine. The sensitivity of berberine and its derivatives could be predicted by 40 out of 3171 proteins. Of them, 29 proteins have been previously involved in drug resistance. Their relationship to berberine and its derivatives is novel. **Conclusions:** Berberine-type compounds may be new candidates against cancer; however, they may develop drug resistance.

## 1. Introduction

Cancer, which caused nearly 10 million deaths in 2020, is one of the leading causes of death globally. Breast, lung, colon, rectum, and prostate cancers are among the prevalent cancer types worldwide. Cancer becomes more destructive if abnormal cells expand beyond their usual borders, i.e., if they form metastases. Cancer is reported to have caused approximately one in six deaths in 2020, emphasizing the importance of the discovery of effective and personalized anticancer drug candidates with minimal side effects, since chemotherapy is still a prominent approach in the management of various types of cancer [1].

Since the early days of cancer management, natural products have held great eminence as drug leads in drug discovery. Prominent examples are the *Vinca* alkaloids, epipodophyllotoxins, taxanes, and their derivatives. Besides, the number of FDA (Food and Drug Administration)-approved natural drug entities has been increasing for the past few decades. To exemplify, 53.3% of the approved 75 small molecules in the area of cancer are of natural origin during a time frame from 1946 to 1980, while it corresponds to 64.9% of the approved 185 small molecules between 1981 and 2020, remarking the rising significance of natural products in cancer therapy [2].

Alkaloids are secondary metabolites broadly distributed in natural sources such as plants and animals. They possess a cyclic ring structure holding one or more basic nitrogen atoms and exhibit potent and ranging activities from antibacterial to anticancer at low concentrations [3,4,5]. Berberine, a benzylisoquinoline alkaloid, occurs specifically in the root, rhizome, and stem bark of numerous medicinal plants such as *Berberis vulgaris* and *Hydrastis canadensis*, and it has various pharmacological activities (e.g., against cancer, cardiovascular, metabolic, and neurological disorders). Berberine has been traditionally used in Ayurvedic and Chinese medicine throughout many centuries for antimicrobial, antiprotozoal, antidiarrheal, and antitrachoma purposes [6,7,8,9].

In addition to its isolation from natural sources, chemical synthesis has been used for the production of berberine at larger scales. Sulfate or chloride salt of berberine is preferred to be applied for clinical intentions, allowing for improved solubility [9,10].

Preclinical and clinical studies have unraveled a range of bioactivities of berberine, including its anticancer potential. Clinical studies on berberine mostly focus on its applications against heart diseases, diabetes, diarrhea, metabolic syndrome, and cancer [11,12,13,14].

Numerous studies have pointed out the anticancer potential of berberine on cancer cell lines with different origins through the modulation of assorted signaling pathways [15,16]. Still, comprising investigations assessing molecular modes of action of berberine on a range of diverse cancer cell lines and compiling comparative data from in silico and in vitro approaches have been quite limited so far. Thus, in the present study, we investigated the influences and mechanisms of berberine in several cancer cell lines from different origins and focused on understanding its molecular mechanisms. Second, we came up with the question of whether the cytotoxicity of berberine was associated with other molecular determinants in the cell line panel of the National Cancer Institute (NCI, Bethesda, MD, USA). Therefore, we executed a proteomic profiling of the NCI cell lines that correlated with the response to berberine. We proposed the molecular modes of action of berberine, based on a holistic view covering the combination of in silico, bioinformatic, and in vitro methods.

## 2. Results

### 2.1. Cytotoxicity of Berberine Chloride as Determined by the Resazurin Reduction Assay

Berberine chloride exhibited cytotoxic activity against a panel of drug-sensitive and -resistant cancer cell lines with the IC_50_ values ranging from 2.13 ± 0.21 µM to 141.75 ± 9.04 µM. Among the tested cell lines, HEK293 and CCRF-CEM cells were most affected by berberine chloride. Based on the IC_50_ values, we calculated the degrees of resistance to berberine chloride (Table 1). P-glycoprotein/ABCB1-expressing CEM/ADR5000 leukemia cells expressed 28.2-fold resistance to berberine chloride compared to their parental drug-sensitive CCRF-CEM cell line. An 18.5-fold cross-resistance was observed in ABCB5-transfected HEK293 cells. BCRP-transfected breast cancer cells were 2.41-fold cross-resistant compared to maternal wild-type cells. While HCT-116 p53 knockout colon carcinoma cells displayed similar sensitivity to berberine chloride as HCT-116 wild-type cells, U87.MG glioblastoma cells transfected with a mutation-activated EGFR cDNA were 3.57-fold resistant to berberine chloride compared with their sensitive, non-transfected counterparts.

### 2.2. Drug Resistance Profiling of Berberine Chloride

Using the NCI panel of cell lines derived from nine different tumor types, we studied whether well-known factors of anticancer drug resistance correlate with the cellular response to berberine chloride. We have chosen ATP-binding cassette transporters (mRNA expression of *ABCB1*, *ABCB5*, *ABCC1*, and *ABCG2*), oncogenes and tumor suppressor genes (mutations in *TP53*, *WT1*, and *NRAS*), other resistance factors (mRNA expression of *HSP90* and *GSTP1*), and markers of cellular proliferation (mRNA expression of *KI67* and *14-3-3*). Anticancer compounds known to inhibit these resistance factors were used as control drugs. The mRNA expression has been determined by microarray hybridization, PCR slot blot, and/or quantitative RT-PCR. The protein expression has been determined by protein arrays or Western blotting, and gene mutations by cDNA sequencing. Functional assays have been used for P-glycoprotein/*ABCB1* (rhodamine 123 accumulation) and TP53 (yeast functional assay). For *ABCB1*, the status of DNA gene amplification has been determined by comparative genomic hybridization arrays. As shown in Table 2, resistance to berberine chloride correlated with *ABCB1*, *EGFR*, and *WT1* but not with other parameters. Statistically significant correlations were found for all control drugs investigated.

### 2.3. Berberine Chloride Uptake Measured by Live Cell Microscopy

Since we found cross-resistance of *BCRP*-transfected MDA-MB-231 BCRP cells but no correlation between *BCRP* mRNA expression and log_10_IC_50_ values for berberine chloride in the NCI cell line panel, we investigated this issue in more detail. We applied live cell microscopy to monitor the drug uptake in living cells.

**Figure 1:**
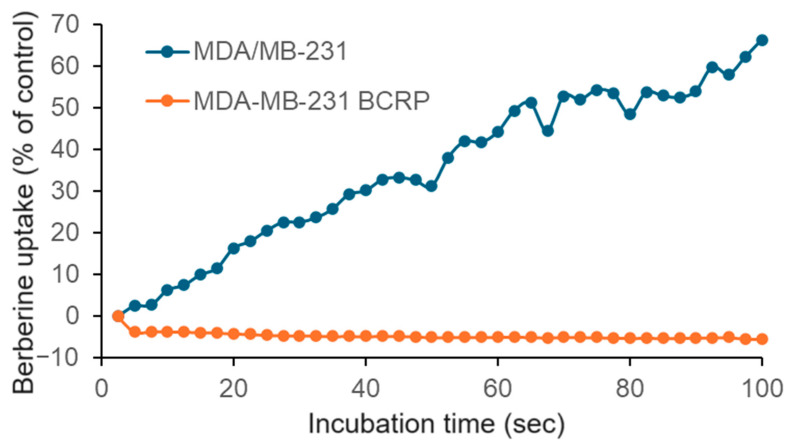
Live cell microscopy of berberine uptake in drug-sensitive MDA-MB-231 and multidrug-resistant MDA-MB-231 BCRP breast cancer cells.

The live cell uptake assay confirmed that berberine chloride entered wild-type MDA-MB-231 cells in a time-dependent manner, while no berberine uptake was observed in the *BCRP*-transfectant cells (Figure 1). Hence, we confirmed the cross-resistance of MDA-MB-231 BCRP cells observed in the resazurin assay by this uptake assay.

### 2.4. Molecular Docking of Berberine to BCRP

**Figure 2:**
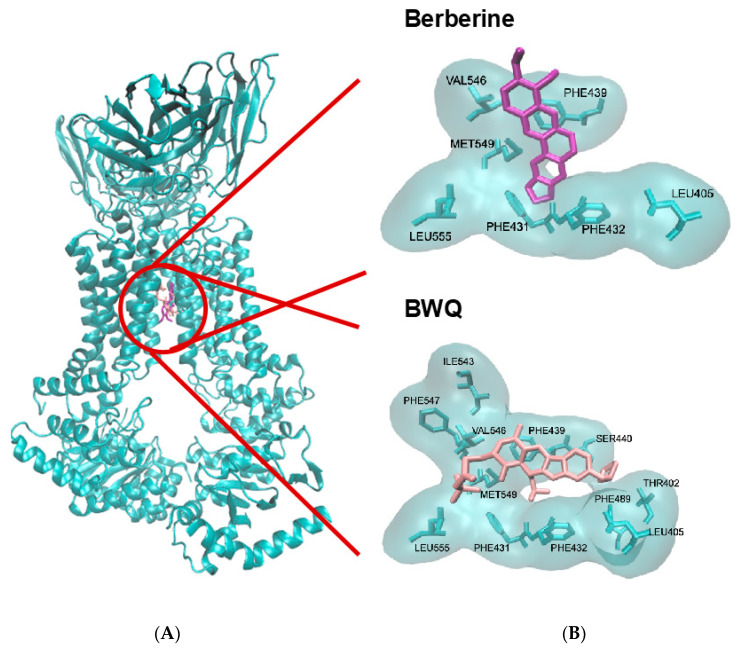
Molecular docking of berberine to BCRP (PDB code 6ETI). (**A**) Berberine interacted with a domain at the inner side of the transmembrane channel formed by BCRP. BWQ was used as a control drug. (**B**) Zoomed view of the binding of berberine and BWQ to BCRP. Berberine interacted with seven amino acids and BWQ with six amino acids, all of which were also in common with berberine. Visual Molecular Dynamics 1.9.3 (VMD) was used for docking visualizations.

To further study the interaction of berberine with BCRP, we performed molecular docking. We used a co-crystallized structure of BCRP bound to its ligand BWQ [17]. We removed BWQ from BCRP and used the unbound BCRP to dock berberine. The results indicated that berberine indeed bound with high affinity to BCRP. The lowest binding energy (LBE) was 8.39 ± 0.01 kcal/mol, the mean binding energy (MBE) was −8.36 ± 0.01 kcal/mol, and the predicted inhibition constant (pK_i_) was 710.09 ± 0.23 nM. The amino acids involved in the interaction were LEU405, PHE431, PHE432, PHE439, VAL546, MET549, and LEU555. All of them displayed hydrophobic interactions (Figure 2A).

As a control, we also docked BWQ to BCRP. As expected, this ligand bound to BCRP with even higher affinity. BWQ displayed an LBE value of −11.43 ± 0.21 kcal/mol, a MBE value of −10.53 ± 0.14 kcal/mol, and a pK_i_ of 4.31 ± 1.33 nM. The amino acids involved in this interaction were VAL401, LEU405, PHE431, PHE439, SER440, VAL546, MET549, LEU555 (Figure 2B). Interestingly, interactions with six amino acid residues were shared by berberine and BQW (LEU405, PHE431, PHE439, VAL546, MET549, and LEU555), implying that both compounds were connected to the same binding domain.

### 2.5. Effect of Berberine Chloride on Microtubules

As described in the literature, berberine inhibits tubulin polymerization [18]. We therefore correlated tubulin expression with the log_10_IC_50_ values for berberine chloride in the NCI cell line panel. However, we did not find a significant correlation. To clarify this contrasting data, we used U2OS cells stably transfected with a cDNA construct of green fluorescent protein (*GFP*) and α-tubulin (*TUBA*) for immunofluorescence microscopy. We treated the cells with berberine chloride as well as with vincristine (a microtubule polymerization inhibitor) and paclitaxel (a microtubule depolymerization inhibitor) as control drugs for 24 h.

**Figure 3:**
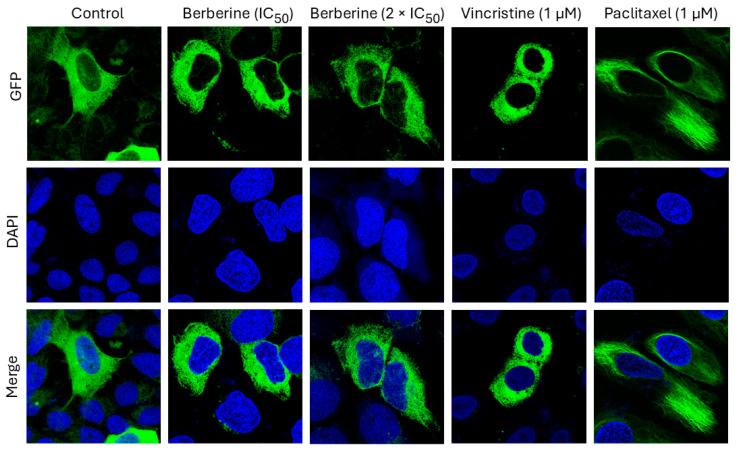
Effect of berberine chloride on the microtubule network in α-tubulin-GFP-transfected U2OS cells. Berberine was used at concentrations of IC_50_ (26.73 µM) or 2 × IC_50_ (53.46 µM). Vincristine (1 µM) and paclitaxel (1 µM) were used as control drugs. The panels show the micrographs of U2OS cells treated for 24 h obtained by AF7000 widefield fluorescence microscope at 63× magnification.

As seen in Figure 3, berberine chloride dose-dependently affected the microtubule formation, adversely. In non-treated cells, the microtubules increasingly spread throughout the cytoplasm and represented an intracellular network. In the case of berberine chloride treatment with IC_50_ or 2 × IC_50_, the mass of the microtubule network lessened specifically at the cell periphery. Their brightness and thickness were reduced in a dose-dependent manner in comparison to non-treated cells, suggesting an inhibitory effect of berberine chloride on microtubule formation.

The morphological changes in the tubulin network in berberine chloride-treated cells were comparable to those seen in vincristine-treated cells but not to those in paclitaxel-treated cells. Vincristine and berberine chloride lessened the enlargement of microtubules at the extremities and enhanced the accumulation of tubulin around the nucleus, unlike paclitaxel-treated cells, in which tubulin seemed rigid. Moreover, the thickness of the microtubules at the peripheries of berberine chloride-treated cells was mitigated in comparison to the negative control cells (Figure 3). This indicates that berberine chloride affected microtubule polymerization rather than microtubule depolymerization.

### 2.6. Prediction of Sensitivity and Resistance to Berberine by Proteomic Profiling

In the first part of our study, we focused on known resistance mechanisms and their relevance for berberine chloride. Now, in the second part, we wanted to identify novel factors that determine either sensitivity or resistance to berberine chloride.

We examined the expression of 3171 proteins in the NCI cell line panel and correlated the expression values to the log_10_IC_50_ values for berberine chloride. We performed Pearson correlation tests to conceive a ranking list of proteins, whose expression is directly or inversely closely associated with the log_10_IC_50_ values for berberine chloride. Only cut-off values of correlation coefficients of r > 0.3 (direct correlations) or r < −0.3 (inverse correlations) were taken into consideration. By this, 40 genes were identified, among which half were directly correlated, and the other half were inversely correlated to the log_10_IC_50_ values for berberine chloride. The proteins encoded by these genes are involved in numerous biological activities, as shown in Table 3.

**Figure 4:**
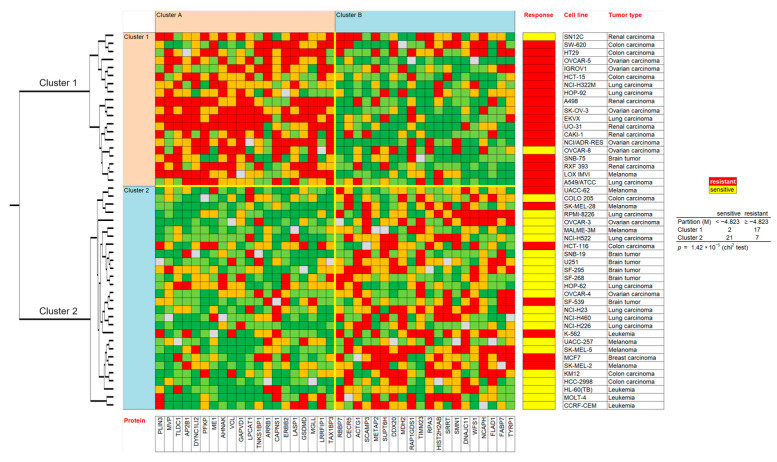
Hierarchical cluster analyses (Ward method) and heatmap of the expression of 40 proteins correlating with the response of 47 tumor cell lines to their log_10_IC_50_ values for berberine chloride. Color code: red and orange protein overexpression, light and dark green protein downregulation compared to the median value (gray).

Then, the expression values of the NCI cell lines for the protein expressions were subjected to hierarchical cluster analysis to determine whether clusters of cell lines may possess predictive power regarding sensitivity or resistance of the cell lines to berberine chloride. The dendrogram of the cluster analysis indicated two main branches in the cluster tree that were represented in the heatmap (Figure 4).

Since the log_10_IC_50_ values for berberine chloride were not previously included in the cluster analysis, they were assigned afterwards to the corresponding position of the cell lines in the cluster tree. The cellular responsiveness to berberine was determined by comparing individual log_10_IC_50_ values to the median value across all cell lines. If the log_10_IC_50_ value was lower than the median, the cell lines were categorized as sensitive. If it was above the median, the cells were defined as resistant to berberine. The distribution among the two clusters was remarkably different from each other (*p* = 1.42 × 10^−5^). Cluster 1 was mostly composed of cell lines resistant to berberine, whereas cluster 2 typically included sensitive cell lines (Figure 4). The median value (−4823 M) of the log_10_IC_50_ values was used as a cut-off value to define the cell lines as being sensitive or resistant to berberine.

### 2.7. Cytotoxicity of Berberine Derivatives

**Figure 5:**
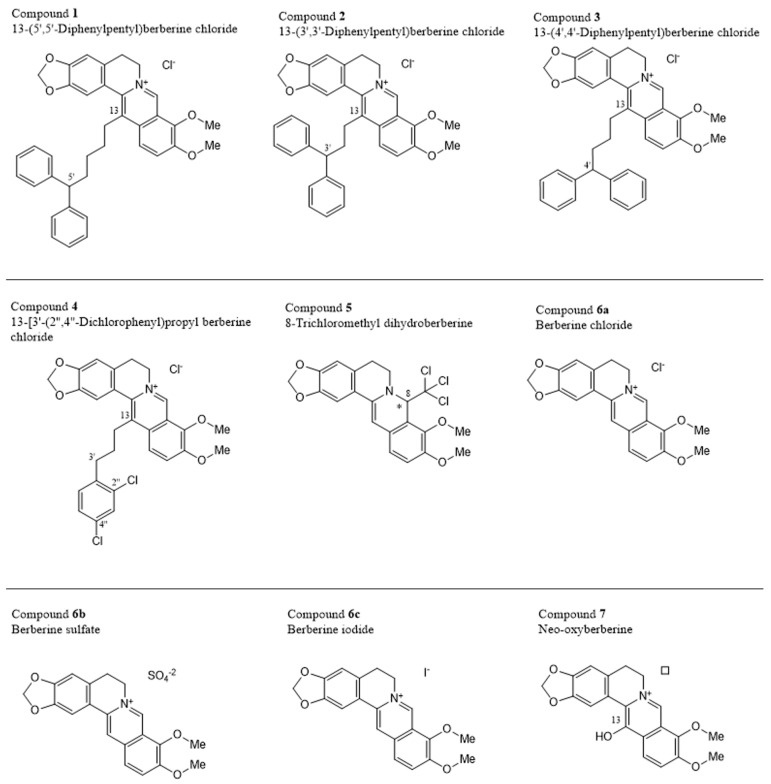
Chemical structures of the investigated berberines * Configuration at the chiral center not indicated, the compound might be racemic. ☐ Counter-anion not indicated by the provider.

In the third part of the current study, we extended our investigations from berberine chloride to eight other berberine derivatives: 13-(5′,5′-diphenylpentyl) berberine chloride (compound **1**), 13-(3′,3′-diphenylpropyl) berberine chloride (compound **2**), 13-(4′,4′-diphenylbutyl) berberine chloride (compound **3**), 13-[3′-(2,4-dichlorophenyl)propyl] berberine chloride (compound **4**), 8-trichloromethyldihydroberberine (compound **5**), and neo-oxyberberine (compound **7**) as drawn in Figure 5. Three of the compounds are salts of berberine, numbered as **6a** (chloride), **6b** (sulfate), and **6c** (iodide). All of these will be converted to berberine once they enter the cells.

**Figure 6:**
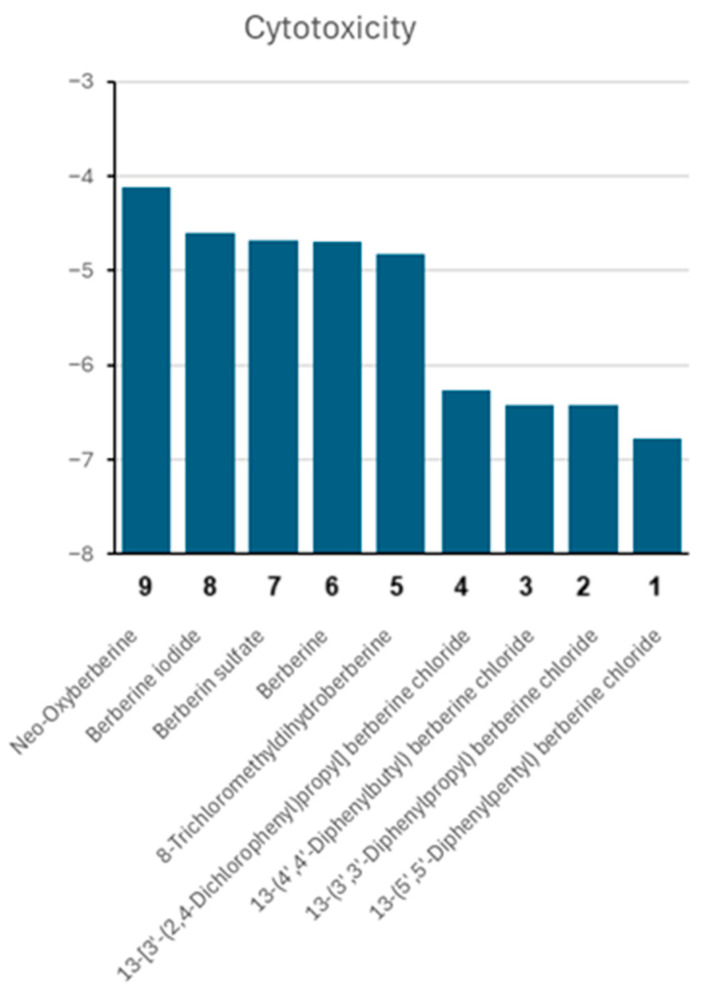
Cytotoxicity of nine berberine-type compounds towards the NCI panel of the NCI, consisting of cell lines derived from different tumor origins (leukemia, melanoma, brain tumors, carcinomas of the prostate, colon, breast, lung, kidney, or ovary). Shown are the mean log_10_IC_50_ values.

We were interested to see the cytotoxic activity of the nine berberine compounds. Therefore, we calculated the mean log_10_IC_50_ values from all 59 NCI tumor cell lines. As shown in Figure 6, compound **1** (13-(5′,5′-diphenylpentyl) berberine chloride) was the most cytotoxic one, followed by **2**, **3**, and **4**, which also displayed high cytotoxicity. Less active were compound **5** and the berberine salts (**6a**, **6b**, and **6c**). Neo-oxyberberine (**7**) was the least active substance in this series.

### 2.8. Structure-Activity Relationships of Berberine Derivatives

The nine investigated compounds consisted of the natural alkaloid berberine as the parent compound, represented as the salts with three different anions, compounds **6a**, **6b**, and **6c**, complemented by a series of berberine analogs, mostly with different substituents at C-13, but also at C-8 in the case of compound **5**. A comparison of the activities of the different berberine salts **6a**, **6b**, and **6c**, with their various counter anions—chloride, sulfate, and iodide, respectively—showed that the counter ions do not play a particular role, as seen from the nearly identical cytotoxicities. However, a drastic increase in activity is observed if attaching a long and bulky, and lipophilic substituent at C-13, as found in compounds **1**–**4**.

In compound **4**, it is a simple monocyclic phenyl ring, extended by the presence of two chlorine substituents, while compounds **1**–**3** have, all of them, two aromatic rings filling the otherwise (i.e., in berberines themselves) free space, in the form of a diphenylmethyl unit. This entity is attached to C-13 of the berberine core by an alkylidene tether, with two to four methylene units between the two molecular portions. While the length of that chain did not have a great impact on the cytotoxicity in compounds **2** (two CH_2_ units) vs. **3** (three CH_2_ chain links), compound **4** (with now four such methylene entities) was again significantly more active. This leads to the assumption that even longer chain lengths might entail even larger activities, before possibly sinking again with too long side chains.

Of particular interest is the case of neo-oxyberberine (**7**), with its additional OH at C-13. This hydroxy group cannot undergo a tautomerization to a non-charged lactam as in the case of oxyberberine itself, so it is an additional, strongly acidic OH group, changing the polarity and H-bridge bond formation capacities as compared to all other derivatives—making it the by far least active among the investigated. Due to its high OH acidity, it might, depending on the local pH value, occur as a zwitterion.

A most unusual analog was compound **5**. It was the only dihydroberberine derivative, thus also the only chiral one (with a stereogenic center at C-8). Furthermore, it was, in particular, the only non-cationic derivative in this series, the nitrogen being part of an electron-rich and basic enamine function, instead of an electrophilic iminium group.

From this, it can be expected to have the most different membrane-permeability properties compared to the other eight cationic derivatives. Of special concern, however, is the presence of a trichloromethyl group at C-8, as also occurring in the insecticide dichlorodiphenyltrichloroethane (DDT), but much more closely related in 1-trichloromethyl-substituted isoquinoline and β-carboline derivatives (e.g., TaClo, an endogenous mammalian neurotoxic alkaloid), whose use should be strictly avoided. Overall, the results show that the by far highest cytotoxicity was observed from derivatives equipped with a large lipophilic diphenylmethane residue, attached to C-13 of the berberine core by a long oligomethylene chain.

### 2.9. Prediction of Sensitivity and Resistance to Berberine Derivatives by Proteomic Profiling

**Figure 7:**
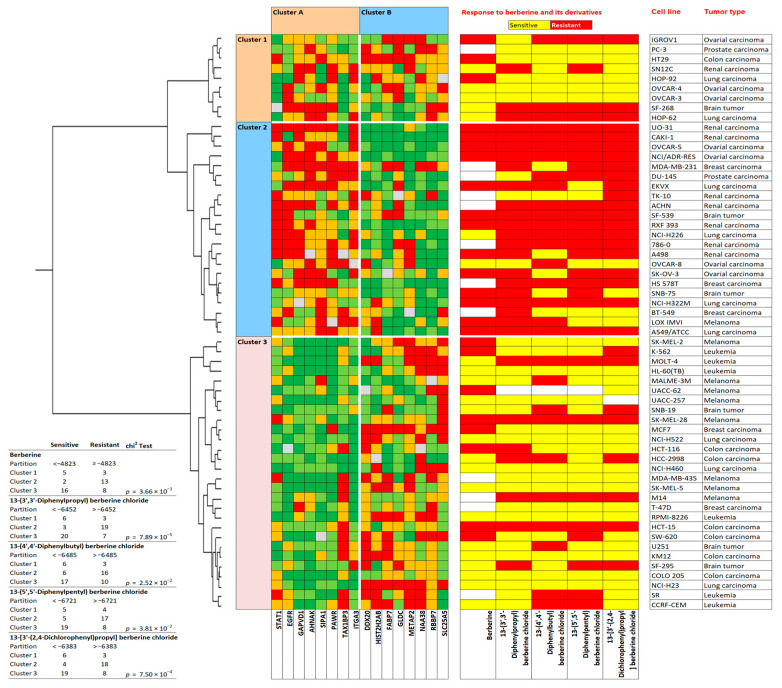
Hierarchical cluster analyses (Ward method) and heatmap of the expression of 16 proteins correlating with the response of 59 tumor cell lines to their log_10_IC_50_ values for five berberine-type compounds (berberine chloride (**6a**), 13-(5′,5′-diphenylpentyl) berberine chloride (**1**), 13-(3′,3′-diphenylpropyl)berberine chloride (**2**), 13-(4′,4′-diphenylbutyl) berberine chloride (**3**), and 13-[3′-(2,4-dichlorophenyl)propyl] berberine chloride) (**4**). Color code: red and orange protein overexpression, light and dark green protein downregulation compared to the median value (gray).

The cell lines clustered in three branches (clusters 1, 2, and 3; Figure 7, left side) and the genes in two branches (clusters A and B; Figure 7, top). Independent of the cluster analysis, we took the log_10_IC_50_ values of berberine chloride (**6a**) as a lead compound and those of the four most cytotoxic representatives, compounds **1**–**4**. The cell lines were categorized as being sensitive and resistant towards these five compounds according to the corresponding median values of their log_10_IC_50_ values. Cell lines that were defined as being resistant were color-coded in red, the sensitive ones in yellow (Figure 7, right side). Cluster 2 contained mainly resistant cell lines, and cluster 3 mainly sensitive ones. Cluster 1 was of a mixed type containing both sensitive and resistant cell lines. Interestingly, 16 genes assembled in clusters A and B predicted cellular responsiveness to all five berberine compounds. The distribution of resistant and sensitive cell lines was significantly different for all five compounds (χ^2^ test; Figure 7). This analysis demonstrated that the profiles of specific genes could be identified that predict sensitivity or resistance to all five berberine-type compounds.

## 3. Discussion

The problem of drug resistance and side effects in oncology. The current paper deals with resistance factors. The development of resistance to cancer drugs has been a major problem in oncology for decades. Therefore, new drugs need to be developed that either circumvent or inhibit the resistance mechanisms to established drugs. Cancer cells can develop various molecular biological mechanisms to become resistant to drugs. These include, for example, reduced accumulation of drugs by the cell membrane and increased metabolism so that lethal drug levels cannot be achieved, gene mutations in drug targets, DNA repair mechanisms, activation of alternative signal transduction pathways and feedback mechanisms, alterations in programmed cell death mechanisms, and many others [19,20,21]. Despite significant progress in drug development, the problem of resistance has become even more acute in recent years. The increasing number of resistance mechanisms and the complexity of cancer biology make it more difficult to develop and apply effective treatments. This is exacerbated by the fact that cancer drugs are not only insufficiently effective due to the development of resistance, but they can also cause severe, sometimes life-threatening side effects that can significantly impair the patients’ quality of life (bone marrow toxicity, nausea, vomiting, fatigue, hair loss, infections, etc.). These side effects occur because cancer drugs not only attack cancer cells, but also healthy proliferating cells. Even new, targeted drugs (small molecules, therapeutic antibodies) are not free of side effects and have not satisfactorily solved the problem of lack of selectivity.

We are therefore looking for new active molecules of natural origin, since many of the cancer drugs that have been established for decades are natural substances and derivatives thereof. The search for bioactive lead structures from nature (medicinal plants, marine organisms, bacterial organisms) is therefore promising; synthetic analogs derived from such substances and pharmacologically improved congeners can be produced synthetically.

One such example is berberine. For ages, berberine-containing plants have been used in traditional Chinese and Ayurvedic medicine. In traditional Chinese medicine (TCM), metabolic disorders, cardiovascular diseases, inflammation, and digestive problems are treated with berberine-rich plants such as *Coptis chinensis* Franch. J. (Ranunculaceae) (golden threadroot), *Phellodendron amurense* Rupr. (Rutaceae) (Amur cork tree), and *Berberis vulgaris* L. (Berberidaceae) (common barberry) [22,23,24]. *Berberis aristata* DC. (Berberidaceae) (Indian barberry) and *Picrorhiza kurroa* Royle ex Benth. (Plantaginaceae) (kutki) are also used in Ayurvedic medicine to alleviate metabolic disorders, inflammation, infections, and liver diseases [25,26,27]. The treatment of cancer is also documented in traditional medicine [28]. Although some mechanisms of the pharmacological effects of berberine have been described in Western pharmacology (e.g., lipid-lowering and insulin-resistance-improving activities), the spectrum of pharmacological effects is much broader, and the molecular mechanisms have not yet been fully investigated [29].

Berberine is generally considered to be safe and exerts very low toxicity in usual doses and reveals clinical benefits without major side effects. Berberine was reported to usually possess a great safety profile in the scientific literature. To exemplify, berberine displayed IC_50_ value above 40 µM in the non-tumor Chang liver cells when treated for 24 h [30]; in addition to having an IC_50_ value above 100 µg/mL in normal human peripheral blood mononuclear cells [31]; an IC_50_ value of 838.4 µM in human normal hepatocyte HL-7702 cell line [32] and an IC_50_ value of 71.14 μg/mL in the normal African green monkey kidney epithelial cell line (Vero) when treated for 48 h [33], all suggesting berberine’s anticancer potential as an efficient and reliable agent. However, some undesirable effects may also occur in some patients, such as gastrointestinal distress, headache, and dizziness. Long-term use might cause liver toxicity, unwanted interactions with other drugs that stabilize blood sugar levels, or heart medications have also become known [34]. Common side effects of berberine consumption include gastrointestinal symptoms, such as constipation and diarrhea [35].

While safety and good tolerability may be true for berberine itself, berberine derivatives may react differently. In this context, compound **5** deserves special attention. It contains a trichloromethyl group at C-8, which also occurs in 1-trichloromethyl-1,2,3,4-tetrahydro-beta-carboline (TaClo). This is an alkaloid that is formed in mammalian organisms by a Pictet-Spengler condensation of endogenous tryptamine (Ta) and the synthetic hypnotic substance chloral (Clo). TaClo has structural similarity to the neurotoxin 1-methyl-4-phenyl-l,2,3,6-tetrahydropyridine (MPTP). TaClo may be involved in the pathogenesis of Parkinson’s disease. Therefore, compound **5** may also be neurotoxic. Its use as a therapeutic drug should therefore be strictly avoided [36,37,38,39].

While the use of berberine-containing plants is well documented in traditional medicine, the question is whether the activity of berberine can also be determined in modern pharmacology. Apart from a plethora of in vitro data [40,41,42], the published data on the anticancer activity of berberine in various tumor types in vivo are promising and may stimulate further research to develop berberine as a novel anticancer drug [43,44,45,46].

*Activity of berberine in vivo and in patients:* In vitro data provide valuable insights into the potential benefits of new drugs. However, translating these findings into clinical practice requires careful pharmacokinetic analyses. Given the fact that berberine is active in animal experiments, the question arises whether pharmacologically active concentrations of berberine can be reached in the blood serum so that clinical effects could be expected in patients. Indeed, berberine can be absorbed and metabolized in the body, reaching levels that may be therapeutically effective. Rats intragastrically administered with berberine showed a bioavailability of 0.37 ± 0.11%. Of the nine metabolites in the blood, more phase II than phase I metabolites were found that were excreted by bile and feces, with a recovery rate of 41.2% [47]. These animal pharmacokinetic data indicate that clinical effects may also occur in patients. A recent meta-analysis of ten randomized clinical trials with a total of 811 patients revealed that the clinical parameters for hepatic enzymes, lipid profile, and insulin sensitivity in patients with non-alcoholic fatty liver disease were significantly improved [48].

Of course, the question now is whether berberine is also active against cancer. A randomized clinical trial with berberine has been performed in patients with refractory colorectal adenoma. While serious adverse effects did not occur, 36% of the 429 berberine-treated patients and 47% of the 462 placebo-treated patients developed recurrent adenoma (*p* = 0.001) [49]. The medicinal plant *Chelidonium majus* L. (Papaveraceae) contains berberine. An extract of *C. majus* (NSC-631570, Ukrain) has been used in a clinical phase II for the palliative treatment of pancreatic cancer, and a doubling of survival time has been observed [50]. However, *C. majus* has also been reported to be hepatotoxic [51]. While we did not find randomized clinical trials on the berberine-containing plants mentioned above regarding cancer, two clinical trials are using a root extract of *C. majus* (termed Ukrain), which contains various alkaloids, including berberine, which has been used to treat pancreatic carcinoma. Remarkably, this extract, in combination with gemcitabine as a standard drug, reached significantly better survival rates than gemcitabine alone [50]. It has to be mentioned, however, that Ukrain has been associated with hepatotoxicity, which is rather due to other constituents such as chelidonine, sanguinarine, and protopin rather than berberine itself [51,52]. At this point, it becomes apparent how necessary it is to perform randomized clinical trials with isolated berberine and berberine-containing plants used in TCM and Ayurveda.

*The role of P-glycoprotein for berberine*: In the past years, we have been running a program on ethnopharmacology to identify new candidates for cancer therapy [53,54,55]. This also raised our interest in berberine as a substrate of the ATP-binding cassette (ABC) transporter, P-glycoprotein. We reported the cross-resistance of multidrug-resistant P-glycoprotein-overexpressing CEM/VCR100 cells that have been selected for vincristine resistance [56] as well as for pancreatic carcinoma cells, a tumor type that is well known to be non-responsive to more or less all kinds of chemotherapy [57]. In the present investigation, we confirm this result for CEM/ADR5000 cells that have been selected for doxorubicin resistance. Berberine has also been reported by others to be a substrate of P-glycoprotein [58,59,60,61]. We also described that plants of the liana *Ancistrocladus tectorius* (Lour.) Merr. (Ancistrocladaceae), which contain berberine-similar alkaloids, display cytotoxicity activity against multidrug-resistant cells [62]. *A. tectorius* is growing in China, but is not commonly used in traditional Chinese medicine. Hence, considering the bioactivity of berberine in the pharmacology of natural products in general, we think that the role of berberine in traditional Chinese medicine, focusing on plants that are typically used in Chinese clinical practice, deserves more attention in the future. Berberine is also a substrate of the ABC-transporter, MRP1 [63].

The role of BCRP for berberine. BCRP-transfected MDA-MB-231-BCRP cells were also cross-resistant to berberine. Using a live cell uptake assay, we confirmed that berberine was taken up in drug-sensitive MDA-MB-231 cells, while no berberine uptake was observed in MDA-MB-231-BCRP cells, confirming the cytotoxicity assay. We exemplarily performed molecular docking to study the interaction of berberine with BCRP. It was interesting that berberine bound to BCRP with higher affinity than the known BCRP ligand BWQ. Berberine has been proposed as an inhibitor of BCRP [64,65]. Inhibitors and substrates both bind to ABC transporters. The decision whether a compound acts as a substrate or as an inhibitor depends on the binding energy. The higher binding energy compounds may act as inhibitors, the lower ones as substrates. It is known that one and the same compound may act both as a substrate if it is combined alone or with an inhibitor that has a higher binding affinity, or it may also act as an inhibitor if it is combined with a substrate with lower binding affinity [66]. Evaluating the molecular docking results showed that berberine has a potent binding affinity via interacting with various amino acids, all of which (LEU405, PHE431, PHE432, PHE439, VAL546, LEU555, and MET549) are also involved in the interaction of BCRP with the known inhibitors in the literature. Remarkably, these amino acids (except for LEU555) are also involved in the crystallized structure of the complex of BCRP and its ligand, BWQ, which are ALA397, VAL401, LEU405, PHE431, PHE432, ASN436, PHE439, SER440, LEU539, THR542, VAL546, MET549, and LEU555 (PDBSum entry 6eti 2025). This indicates that these amino acids may be crucial for binding and may render a model for forthcoming drug leads of BCRP inhibitors.

Nevertheless, it should be taken into mind that most—if not all—natural products act in a multi-specific manner by binding rather to several than to single targets [67]. This is also true for berberine, which does not only bind to BCRP but also to signal transduction proteins (AMP-activated protein kinase (AMPK), phosphatidylinositol-4,5-bisphosphate 3-kinase catalytic subunit α (PIK3CA)), transcription factors (mammalian target of ra-pamycin (mTOR), nuclear factor erythroid 2-related factor 2 (Nrf2)), regulation of met-abolic processes (sirtuin 1 (SIRT1)), antioxidant response, and detoxification (forkhead box O1 (FOXO1), glutathione peroxidase (Gpx) 4, and NAD(P)H quinone oxidoreductase 1 (NQO1)), all of which are involved in the regulation of cell growth, cancer, inflammation, metabolism, and oxidative stress [68,69,70,71]. These interactions contribute to the therapeutic potential of berberine in treating various conditions, including metabolic disorders, inflammation, and oxidative stress.

The role of tumor suppressor genes and oncogenes for berberine. Tumor suppressor genes and oncogenes are not only relevant for carcinogenesis and tumor progression but also determine drug resistance [72]. The most well-known tumor suppressor is p53. It maintains the integrity of DNA by controlling cell cycle arrest, DNA repair, and apoptosis. We found that p53 wild-type and knockout cells were similarly inhibited by berberine, indicating that p53-dependent and -independent mechanisms exist by which berberine can exert its cytotoxicity. Comparable observations have been reported by others [73,74,75,76,77].

Epidermal growth factor receptor (EGFR), a transmembrane tyrosine kinase receptor, functions in cell survival, growth, and division. Amplification of and mutations in the EGFR gene are among the main players in the occurrence of various human cancer cells and poor prognosis of cancers [78,79,80].

While the tumor suppressor p53 did not influence the cellular responsiveness to berberine, we found that cells expressing the mutation-activated oncogene EGFR were more resistant to berberine than wild-type cells. This result is congruent with data from other authors [81,82]. We have previously reported that EGFR can confer resistance to a broad range of established anticancer drugs and has prognostic significance for the survival of cancer patients [83,84]. The results of the present investigation extend this list to berberine.

The Wilms’ tumor gene (WT1) encodes a zinc finger transcription factor involved in tissue development, in cell proliferation and differentiation, and in apoptosis, and is categorized as a tumor suppressor gene. The WT1 proteins serve as transcription factors but may also participate in splicing. Interruption of these actions may cause abnormal growth [85]. Our research further unraveled the relationship between the log_10_IC_50_ values of berberine and EGFR and WT1 expressions, indicating that berberine may be affected by both EGFR and WT1 proteins, contributing to the development of resistance.

The role of tubulin for berberine. We also addressed the question, whether or not berberine inhibits tubulin. The proteome-based correlation analyses did not reveal an association between berberine and tubulin. However, the log_10_IC_50_ values of berberine correlated with several microtubule-associated proteins (dynein cytoplasmic 1 light intermediate chain 2, vinculin, LIM and SH3 domain protein 1, and actin γ1), which may further be a clue to the impact of berberine on the microtubule cytoskeleton. Therefore, we wanted to clarify the action of berberine on microtubule dynamics. Berberine-treated U2OS cells expressing α-tubulin-GFP were monitored by widefield fluorescence microscopy. Our results indicated that berberine inhibits tubulin polymerization (like vincristine) but not depolymerization (like paclitaxel). There are also hints in the literature that berberine interacts with tubulin. Berberine moderately arrested the cell cycle in the G2/M phase of the cell cycle, and berberine bound to isolated tubulin from goat brain [18]. The authors found that berberine binds to the colchicine binding site of tubulin. This data fits our results because colchicine is also a polymerization inhibitor. The berberine derivative, oxyepiberberine, has also been described as a tubulin polymerization inhibitor [86].

Identification of novel determinants of cellular responsiveness towards berberine by proteome analysis. The proteome-based approach applied by us in the present investigation has been frequently used in the past years to get hints at the molecular mechanisms of cytotoxic compounds and novel anticancer drugs [87,88]. It has initially been described as a transcriptomics-based pattern recognition analysis by the National Cancer Institute (USA) [89]. It is a striking feature of tumors that their responsiveness to anticancer drugs can be determined by their molecular architecture. The NCI cell line panel, consisting of nine tumor types (leukemia, melanoma, CNS tumors, and carcinomas of lung, colon, breast, prostate, ovary, and kidney) does not only reflects the sensitivity or resistance of the corresponding tumor types to tested drugs but also the mode of action by using a COMPARE analysis based on the Pearson correlation test. The Developmental Therapeutics Program of NCI tested more than 200,000 compounds using this cell line panel and contributed to the identification and development of novel anticancer drugs.

We used the NCI repository to investigate berberine and its derivatives. We correlated the expression of 3171 proteins with the log_10_IC_50_ values of berberine and identified 40 proteins with the best correlation coefficients. The modes of action of drug, resistance, and sensitivity determinants may be foreseen by the patterns of drug activity across the NCI cell lines. The expression values of these proteins were used to perform hierarchical cluster analyses to generate two-dimensional color-coded heat maps, where in one dimension, the expression profiles of the 40 proteins appear, and in a second dimension, the different cell lines appear. The methodology based on proteomic expression was linked to the activity of berberine. The cellular responsiveness of berberine was associated with proteins with diverse functions such as cancer development and metastasis (AHNAK, LPCAT1, ERBB2, GSDMD, MGLL, TAX1BP3, METAP2), chromosomal function (RBBP7, SUPT6H, HIST2H2AB, NCAPH), cytoskeleton (DYNC1LI2, VCL, LASP1, ACTG1), mitochondrial function (DDX20, MDH2, RAP1GDS1, TIMM23, DNAJC11), metabolism (PLN3, PFKP, ME1, CECR5, FLAD1, FABP7), transport function (MVP, GAPVD1, SCAMP3), DNA repair (TNKS1BP1, RPA3), and others (TLDC1, AP2B1, ARRB1, CAPNS1, LRRFIP1, SRRT, SMN1, WFS1, TYRP1). The results shown in Table 3 strengthen the hypothesis that berberine affects crucial cellular functions in tumor cells.

Although these proteins were not described as resistance factors for berberine as of yet, it is interesting that 29 out of these 40 proteins have been recently described as resistance factors to standard anticancer medications (Table 4). This can be taken as a clue that our proteome-based analysis identified proteins that are relevant for drug resistance of tumors in general, including berberine-type drugs.

Role for drug sensitivity prediction. Our proteome analysis not only provided clues on the cytotoxic mechanisms of berberine-tape compounds towards cancer cells. The estimation of the response of cancer cells to a cytotoxic agent based on their protein expression profiles is a major focus in precision medicine, because the sensitivity or resistance of cancer cells towards drug-candidate molecules like berberine may be foreseen by such a bioinformatic approach. In principle, it can be imagined to determine the sensitivity to established anticancer drugs and also investigational drugs like berberine in human tumor biopsies before chemotherapy to predict the drug sensitivity or resistance of individual tumors to specific drugs. This information may be used to individually adapt the drug regimen for each tumor. Hence, determining the molecular architecture of individual tumors may be a step forward to individualized tumor treatment and precision medicine in oncology, because tumors resistant to standard chemotherapy may still be sensitive to these compounds.

These cluster image maps can be used to monitor the response of tumors to chemotherapy. Protein (or gene) expression profiles from cluster image maps may be helpful to create personalized treatment plans, enabling more precise and effective therapies. Protein expression signatures and biomarkers derived from cluster image maps can also be instrumental in predicting the prognosis of patients after chemotherapy.

In the future, new target discovery technologies such as Proteolysis-Targeting Chimera (PROTAC) probe technology and proteomic techniques are supposed to lead the discovery of additional targets and mechanisms of natural products like berberine and its derivatives. With the assistance of artificial intelligence-driven algorithms, complex interactions between natural products and target proteins may be predicted, highlighting their molecular mechanisms on these proteins. Furthermore, PROTAC may head the disclosure of undruggable entities and their targeted degradation, providing innovative therapeutic paths for natural drug development [137,138,139,140].

## 4. Materials and Methods

### 4.1. Cell Lines

The cell lines used in the present work, their origins, and maintenance conditions were previously reported [54,141]. In brief, MDA-MB-231 breast cancer cells and their transfectant subline MDA-MB-231-BCRP clone 23 were a generous gift from Dr. Douglas D. Ross (University of Maryland Greenebaum Cancer Center, University of Maryland School of Medicine, Baltimore, MD, USA), HCT116 (p53^+/+^) colon cancer cells and its knockout clone HCT116 (p53^–/–^) were kindly provided by Dr. B. Vogelstein and H. Hermeking (Howard Hughes Medical Institute, Baltimore, MD, USA), U87.MG glioblastoma multiform cells and their transfectant subline U87.MGΔEGFR were obtained from Dr. W.K. Cavenee (Ludwig Institute for Cancer Research, San Diego, CA, USA) as well as HEK293 human embryonic kidney cells transfected with or without a cDNA for ABCB5, a generous gift from Prof. Yoshikazu Sugimoto (Division of Chemotherapy, Faculty of Pharmacy, Keio University, Tokyo, Japan), and wild-type human osteosarcoma U2OS cells were used.

U2OS human osteosarcoma cancer cells stably transfected with an α-tubulin-GFP construct were obtained from Dr. Joachim Hehl (Light Microscopy Centre, ETH Zürich, Zürich, Switzerland). The cells were cultured in DMEM medium (Invitrogen, Darmstadt, Germany) with 10% FBS (A5670701, Thermo Fisher Scientific, Waltham, MA, USA) and 1% penicillin (100 U/mL)-streptomycin (100 μg/mL) (PIS) antibiotic (10378016, Thermo Fisher Scientific, Waltham, MA, USA) and continuously treated with 250 μg/mL geneticin (10131035, Thermo Fisher Scientific, Waltham, MA, USA) at 37 °C and 5% CO_2_ to maintain α-tubulin expression as previously described [142].

A panel of 60 human tumor cell lines was previously described [143] and used by the Developmental Therapeutics Program of the National Cancer Institute (NCI, Bethesda, MD, USA) for drug screening purposes. The cell lines were of diverse origin, including leukemia, melanoma, brain tumors, and carcinoma of the lung, colon, kidney, ovary, breast, or prostate. We have excluded one cell line from this panel (MDA-N) because it was initially misclassified as breast cancer but later identified as a melanoma cell line derived from MDA-MB-435 cells [144]. Hence, we use only 59 cell lines of the NCI tumor panel in the present study. The results of the drug screening (log_10_IC_50_ values obtained by a sulforhodamine 123 assay) as well as transcriptomic and proteomic expression data were deposited at the NCI website (https://dtp.cancer.gov).

### 4.2. Resazurin Reduction Assay

The cell viability of tumor cells under the treatment of berberine chloride (B3251-5g, Sigma-Aldrich, Taufkirchen, Germany; purity ≥ 98%) was evaluated using the resazurin reduction assay. The procedure was previously described [145,146]. Berberine chloride was applied to the tumor cells at various concentrations ranging from 0.003 to 100 μM. Each assay was independently performed thrice with six parallel replicates each. Dose–response curves of each cell line were formed using Microsoft Excel (Microsoft^®^ Version 16.77.1, 2023).

### 4.3. Hierarchical Cluster Analyses of Proteomic Expression Profiles

We performed the Pearson correlation test and hierarchical cluster analyses using the mass-spectrometry-based proteomic expression data of 59 cell lines from NCI (https://dtp.cancer.gov) to produce a rank-ordered list comprising the top 20 proteins that directly and the top 20 proteins that inversely correlated with the resistance of berberine chloride, based on the log_10_IC_50_ values of the cell lines. A heat map relying on the agglomerative clustering according to the Ward method was formed. The method was previously applied by our group members [54,142]. To investigate the association of these candidate proteins with further berberine derivatives, the top 8 up- and downregulated protein expression profiles have been subjected to a second cluster analysis. The intention was to see whether these proteins are also of more general predictive value for the sensitivity or resistance of tumor cell lines to berberine-type compounds.

### 4.4. Molecular Docking with AutoDock 1.5.7

We performed molecular docking via AutoDockTools-1.5.7 for the estimation of the probable interaction of berberine chloride with ATP-binding cassette sub-family G member 2 (breast cancer resistant protein, ABCG2, BCRP). In this respect, we selected the three-dimensional structure of human ATP-binding cassette sub-family G member 2 as a target protein and obtained it from the Protein Data Bank (PDB) (https://www.rcsb.org, PDB ID:6eti) [17]. The resolution of the crystal structure was 3.1 Å. This structure was co-crystallized with the specific ABCG2 ligand tert-butyl3-((3S,6S,12aS)-9-(cyclopentyloxy)-6-isobutyl-1,4-dioxo-1,2,3,4,6,7,12,12a-octahydro-pyrazino [1′,2′:1,6]pyrido[3,4-b]indol-3-yl)propanoate (BWQ; also known as MZ29). This ligand was removed from the co-crystallized structure, and the ABCG2 protein was then used for molecular docking with berberine and berberine chloride. The 3D molecular structures of the ligands (berberine and berberine chloride) were created by using their SMILES from PubChem (https://pubchem.ncbi.nlm.nih.gov), followed by transforming them into Protein Data Bank (pdb) files via CORINA Classic (https://demos.mn-am.com/corina_interactive.html, accessed on 15 December 2025) or NovoPro (https://www.novoprolabs.com/tools/smiles2pdb, accessed on 15 December 2025). Subsequently, these PDB files were converted to PDBQT (Protein Data Bank partial charge and atom type) files. The grid box was created based on the crystallized structure of the known inhibitor-bound ABCG2 available in the literature (the amino acids involved in inhibitor ligand-ABCG2 interaction were taken into consideration during the formation of the grid box that covered these residues). The Lamarckian algorithm, calculating 250 runs and 25,000,000 energy evaluations for each cycle, was used in docking analysis. Docking log (dlg) files, containing the lowest binding, the mean binding affinity, and the predicted inhibition constant (pKi), rendered the required information about docking outcomes. The amino acids involved in the interaction of ABCG2 with berberine and berberine chloride were described via AutoDockTools. Visual Molecular Dynamics 1.9.3 (VMD) was used for docking visualizations (http://www.ks.uiuc.edu/Research/vmd/, accessed on 15 December 2025).

### 4.5. Berberine Uptake by Live-Cell Microscopy

We performed a berberine uptake assay to study whether berberine is a substrate of BCRP. For this purpose, MDA-MB-231 breast cancer cells and their transfectant subline MDA-MB-231-BCRP clone 23 were treated with 1 µM berberine. Image series were automated in time intervals of 2.5 s with the Improvision Openlab system (versions 3.1.4, 3.1.7, or 3.5.2) in brightfield and fluorescence mode (Ex *; Em *) with an Axiovert S100TV inverted microscope (Zeiss, Oberkochen, Germany), equipped with an LD Achromat 20× 0.4 objective and a Hamamatsu CCD C4742-95 camera (Hamamatsu Photonics Deutschland GmbH, Herrsching am Ammersee, Germany). With increasing fluorescence intensity, the first image was used as the background image, and a background value was subtracted from all subsequent images. Descending image sequences were not processed. After conversion into TIF format, the gray values of the fluorescence images were analyzed with the NIH software Image J134 (https://imagej.net) and displayed graphically with the SigmaPlot software https://sigmaplot.en.softonic.com/ (version 13, Systat Software GmbH, Frankfurt, Germany). This experiment was enabled by the fact that berberine chloride is a naturally yellow-colored compound, whose cellular uptake can be observed by live cell microscopy.

### 4.6. Imaging of Structure and Dynamics of the Microtubule Cytoskeleton by Fluorescence Microscopy

U2OS human osteosarcoma cells transfected with an α-tubulin-GFP construct (30,000 cells/well) were seeded in a µ-Slide 8-Well (ibidi, Gräfelfing, Germany). The cells were provided by Dr. Joachim Hehl (Light Microscope Center, ETH Zürich, Zürich, Switzerland). The cells were allowed to attach overnight in a 37 °C incubator with 5% CO_2_. Then, the cells were treated with IC_50_ and 2 × IC_50_ values of berberine chloride on wild-type U2OS cells. DMSO was used as a negative control. Vincristine (1 µM) was used as a positive control that obstructs polymerization, and paclitaxel (1 µM) was used as a positive control that prevents depolymerization. Both Vincristine and paclitaxel were kindly provided by the Johannes Gutenberg University (University Medical Center of Mainz, Germany). Following berberine chloride treatment for 24 h, the cells were washed with PBS and fixed with 4% paraformaldehyde for 15 min. Subsequently, the cells were washed twice with PBS, and their nuclei were stained with 4′,6-diamidino-2-phenylindole DAPI (1 µg/mL) (28718-90-3, Sigma-Aldrich, Darmstadt, Germany) for 5 min at room temperature. In the following, the cells were washed with PBS twice to remove non-specific staining. The cells were then mounted with ibidi mounting medium (50001, ibidi, Gräfelfing, Germany) and visualized with an AF7000 widefield fluorescence microscope (Leica Microsystems, Wetzlar, Germany). GFP and DAPI were excited with the blue laser (470 nm). GFP emitted light at 525 nm; however, DAPI emitted light at 447 nm. Finally, the fluorescent images were analyzed using Fiji ImageJ software (ImageJ2, Version 2.14.0/1.54f) (National Institutes of Health, Bethesda, MD, USA). The method was previously performed by our group [54].

## 5. Conclusions

In conclusion, cell lines with classical drug resistance mechanisms, such as ABC transporters (ABCB1, BCRP) and tumor suppressors and oncogenes (EGFR, WT1), displayed cross-resistance to berberine chloride. P-glycoprotein/ABCB1, EGFR, and WT1 expression were linked to the log_10_IC_50_ values for berberine in the NCI cell line panel. Hierarchical cluster analyses of proteomic data of the NCI cell line panel revealed a number of further proteins that may be involved in the sensitivity or resistance of tumor cell lines towards berberine-type compounds. Their relationship to berberine and other berberine derivatives is novel. Berberine-type compounds may be new candidates for anticancer drug development. However, they may also be prone to the development of drug resistance.

## Figures and Tables

**Table 1:** IC_50_ values of berberine chloride towards drug-sensitive and multidrug-resistant cell lines using the resazurin assay. Cell lines overexpressing ATP-binding cassette transporters (*ABCB1*, *ABCB5*, *ABCG2*), tumor suppressors, and oncogenes (*EGFR*) or knocked out in tumor suppressors (*TP53*) and their corresponding counterpart cell lines have been investigated. The IC_50_ values were calculated from dose–response curves and represent the mean ± SD of three independent experiments with each six parallel measurements. The degrees of resistance have been calculated by dividing the IC_50_ values of resistant cell lines by the IC_50_ values of the corresponding sensitive cell lines.

Gene	Cell Line	IC_50_ (µM)	Degree of Resistance
*ABCB1*	CCRF-CEM	5 ± 1	28.2
	CEM/ADR5000	141 ± 9	
*ABCB5*	HEK293	2 ± 0	18.5
	HEK293-ABCB5	37 ± 3	
*ABCG2*	MDA-MB-231	17 ± 1	2.41
	MDA-MB-231-BCRP	41 ± 7	
*TP53*	HCT-116 p53^+/+^	19 ± 1	0.94
	HCT-116 p53^–/–^	18 ± 0	
*EGFR*	U87.MG	7 ± 0	3.57
	U87.MGΔEGFR	25 ± 13	

**Table 2:** Relationships between the responsiveness of tumor cell lines of the NCI panel to berberine chloride and known mechanisms of drug resistance, viz., ABC transporters (ABCB1, ABCB5, ABCC1, and ABCG2), oncogenes and tumor suppressors (*TP53*, *WT1*, and *NRAS*), other resistance factors (HSP90 and GSTP1), as well as cell proliferation markers (KI67 and 14-3-3). * *p*-value < 0.05 and r > 0.3 (direct correlations) or r < −0.3 (inverse correlations).

Drug Resistance Mechanism	*p*- or *r*-Value	Berberine Chloride (log_10_IC_50_, M)	Control Drug (log_10_IC_50_, M)
*ABCB1* Expression			Epirubicin
7q21 (Chromosomal	*r*-value	* 0.389	* 0.447
Locus of *ABCB1* Gene)	*p*-value	* 0.005	* 3.55 × 10^−4^
*ABCB1* Expression	*r*-value	* 0.531	* 0.588
(Microarray)	*p*-value	* 5.15 × 10^−5^	* 0.588.82 × 10^−6^
*ABCB1* Expression	*r*-value	*0.476	* 0.410
(RT-PCR)	*p*-value	* 4.15 × 10^−4^	* 1.54 × 10^−3^
Rhodamine 123	*r*-value	* 0.553	* 0.526
Accumulation	*p*-value	* 1.86 × 10^−5^	* 1.12 × 10^−5^
*ABCB5* Expression			Maytansine
*ABCB5* Expression	*r*-value	−0.098	* 0.454
(Microarray)	*p*-value	0.258	* 6.67 × 10^−4^
*ABCB5* Expression	*r*-value	−0.184	* 0.402
(RT-PCR)	*p*-value	0.109	* 0.0026
*ABCC1* Expression			Vinblastine
DNA Gene	*r*-value	0.294	* 0.429
Copy Number	*p*-value	0.021	* 0.001
*ABCC1* Expression	*r*-value	0.296	* 0.398
(Microarray)	*p*-value	0.022	* 0.003
*ABCC1* Expression	*r*-value	0.124	0.299
(RT-PCR)	*p*-value	0.196	* 0.036
*ABCG2* Expression			Pancratistatin
*ABCG2* Expression	*r*-value	−0.011	* 0.323
(Microarray)	*p*-value	0.471	* 0.006
ABCG2 Expression	*r*-value	−0.045	* 0.346
(Western Blot)	*p*-value	0.382	* 0.004
*EGFR* Expression			Erlotinib
*EGFR* Expression	*r*-value	0.298	* −0.458
(Microarray)	*p*-value	0.021	* 1.15 × 10^−4^
*EGFR* Expression	*r*-value	* 0.336	* −0.379
(PCR Slot Blot)	*p*-value	* 0.011	* 0.002
EGFR Expression	*r*-value	0.292	* −0.376
(Protein Array)	*p*-value	0.023	* 0.002
*WT1* Expression			Melphalan
*WT1* Expression	*r*-value	* −0.319	* −0.346
(Microarray)	*p*-value	* 0.014	* 0.004
*TP53* Mutation			5-Fluorouracil
*TP53* Mutation	*r*-value	0.024	* −0.502
(cDNA Sequencing)	*p*-value	0.450	* 3.50 × 10^−5^
TP53 Function	*r*-value	0.144	* −0.436
(Yeast Functional Assay)	*p*-value	0.175	* 5.49 × 10^−4^
*NRAS* Mutation			Doxorubicin
Codon 12 mutation	*r*-value	−0.16071	* −0.424
(cDNA Sequencing)	*p*-value	0.155	* 9.61 × 10^−4^

**Table 3:** Top 40 out of a total of 3171 proteins correlating directly or inversely with the log_10_IC_50_ values of the NCI tumor cell line panel for berberine chloride.

Code	Name	Function	Category
PLIN3	Perilipin 3	Mannose 6-phosphate receptor required for endosome-to-Golgi transport	Metabolism
MVP	Major vault protein/lung resistance-related protein	Multi-subunit ribonucleoprotein structures involved in nucleo-cytoplasmic transport	Transport function
TLDC1	MTOR-associated protein, Eak-7 homolog	TOR signaling, positive regulation of protein localization to the lysosome	Others
AP2B1	Adaptor-related protein complex 2 subunit β1	Links clathrin to receptors in coated vesicles.	Others
DYNC1LI2	Dynein cytoplasmic 1 light intermediate chain 2	Microtubule-associated motor protein	Cytoskeleton
PFKP	Phosphofructokinase, platelet	Glycolysis regulation	Metabolism
ME1	Malic enzyme 1	Generates NADPH for fatty acid biosynthesis; links the glycolytic and citric acid cycles	Metabolism
AHNAK	Desmoyokin	Structural scaffold protein involved in metastasis	Cancer development and metastasis
VCL	Vinculin	Cytoskeletal protein associated with cell–cell and cell–matrix junctions; involved in anchoring F-actin to the membrane	Cytoskeleton
GAPVD1	GTPase-activating protein and VPS9 domain-containing protein 1	Regulation of protein transport	Transport function
LPCAT1	Lysophosphatidylcholine acyltransferase 1	Phospholipid metabolism; involved in tumor progression	Cancer development and metastasis
TNKS1BP1	Tankyrase-1-binding protein	Double-strand break repair and regulation of protein phosphorylation	DNA repair
ARRB1	Arrestin β1	Agonist-mediated desensitization of G-protein-coupled receptors; regulation of receptor-mediated immune functions	Others
CAPNS1	Calpain small subunit 1	Calcium-dependent cysteine proteinase; involved in apoptosis, proliferation, migration, adhesion, and autophagy	Others
ERBB2	V-Erb-B2 avian erythroblastic leukemia viral oncogene homolog 2	Oncogene; regulation of proliferation	Cancer development and metastasis
LASP1	LIM and SH3 domain protein 1	cAMP and cGMP-dependent signaling protein that binds to the actin cytoskeleton at extensions of the cell membrane	Cytoskeleton
GSDMD	Gasdermin D	Tumor suppressor; regulation of epithelial proliferation	Cancer development and metastasis
MGLL	Monoglyceride lipase	Serine hydrolase, role in carcinogenesis and metastasis	Cancer development and metastasis
LRRFIP1	Leucine-rich repeat flightless- interacting protein 1	DNA-binding transcription repressor activity	Others
TAX1BP3	Tax1 (human T-cell leukemia virus type I) binding protein 3	Promotes protein–protein interactions that affect cell signaling, adhesion, protein scaffolding, and receptor and ion transporter functions; involved in metastasis	Metastasis
RBBP7	Retinoblastoma binding protein 7, chromatin remodeling factor	Regulation of cell proliferation and differentiation	Chromosomal function
CECR5	Haloacid dehalogenase-like hydrolase domain containing 5	Involved in glycerophospholipid biosynthesis	Metabolism
ACTG1	Actin γ1	Cell motility and maintenance of the cytoskeleton	Cytoskeleton
SCAMP3	Secretory carrier membrane protein 3	Carrier to the cell surface in post-Golgi recycling pathways; protein trafficking in endosomal pathways	Transport function
METAP2	Methionyl aminopeptidase 2	Methionyl aminopeptidase protects the α subunit of eukaryotic initiation factor 2 from inhibitory phosphorylation; involved in cancer	Cancer development and metastasis
SUPT6H	Suppressor of Ty 6 (SPT6) homolog (*S. cerevisiae*)	Regulation of transcription elongation by RNA polymerase II and transcription elongation-coupled chromatin remodeling	Chromosomal function
DDX20	DEAD/H (Asp-Glu-Ala-Asp/His)Box helicase 20	Putative RNA helicase involved in translation initiation, nuclear and mitochondrial splicing, ribosome and spliceosome assembly, regulation of cell growth and division	Mitochondrial function
MDH2	Malate dehydrogenase 2, mitochondrial	Oxidation of malate to oxaloacetate; role in the malate-aspartate shuttle that operates in the metabolic coordination between cytosol and mitochondria	Mitochondrial function
RAP1GDS1	Rap1 GTPase-GDP dissociation stimulator 1	Stimulatory GDP/GTP exchange protein; regulates mitochondrial dynamics	Mitochondrial function
TIMM23	Translocase of the inner mitochondrial membrane 23	Transport of transit peptide-containing proteins across the inner mitochondrial membrane	Mitochondrial function
RPA3	Replication protein A3	DNA repair and DNA replication	DNA repair
HIST2H2AB	Histone cluster 2 H2A family member B	Replication-dependent histone responsible for the nucleosome structure of the chromosomal fiber	Chromosomal function
SRRT	Serrate RNA effector molecule homolog (*Arabidopsis*)	Involved in primary miRNA processing	Others
SMN1	Survival of motor neuron 1, telomeric	The coding gene is part of a 500 kb inverted duplication on chromosome 5q13.	Others
DNAJC11	DnaJ heat shock protein family (Hsp40) member C11	Involved in cristae formation	Mitochondrial function
WFS1	Wolframin ER transmembrane glycoprotein	Regulation of cellular Ca^2+^ homeostasis in the endoplasmic reticulum	Others
NCAPH	Non-SMC condensin I complex subunit H	Involved in the conversion of interphase chromatin into condensed chromosomes; associated with mitotic chromosomes	Chromosomal function
FLAD1	Flavin adenine dinucleotide synthetase 1 homolog (*S. cerevisiae*)	Catalyzes the adenylation of flavin mononucleotide (FMN) to form flavin adenine dinucleotide (FAD) coenzyme	Metabolism
FABP7	Fatty acid-binding protein 7	Binds long-chain fatty acids	Metabolism
TYRP1	Tyrosinase-related protein 1	Role in the melanin biosynthetic pathway	Others

**Table 4:** Involvement of proteins identified by our proteome analysis (Figure 4) in the responsiveness of tumors to anticancer drugs.

Protein	Involved Drugs	Tumor Type	Reference
PLIN3	Docetaxel	Prostate Ca	[90]
	Sunitinib	Renal clear cell Ca	[91]
MVP			
AP2B1	Erlotinib	Non-small cell lung cancer	[92]
	Cisplatin	Ovarian Ca	[93]
PFKP	Gefitinib	Lung adenocarcinoma	[94]
	Rotenone, navitoclax, and orlistat	Chronic lymphocytic leukemia	[95]
	Sunitinib	Renal clear cell Ca	[96]
ME1	Gefitinib	Non-small cell lung cancer	[97]
AHNAK	Doxorubicin	Breast Ca	[98]
	Paclitaxel, docetaxel, erlotinib, everolimus, and dasatinib	Diverse	[99]
VCL	Doxorubicin	Breast Ca	[100]
	Trastuzumab	Breast Ca	[101]
LPCAT1	Paclitaxel	Breast Ca	[102]
TNKS1BP1	Bevacizumab	Ovarian Ca	[103]
ARRB1	Cisplatin, etoposide	Non-small cell lung cancer	[104]
	Gemcitabine	Bladder Ca	[105]
	Imatinib	Chronic myeloid leukemia	[106]
CAPNS1	Cisplatin	Gastric cancer	[107]
ERBB2	Trastuzumab	Breast Ca	[108]
LASP1	Cisplatin	Esophageal squamous cell Ca	[109]
	Cisplatin	Non-small cell lung cancer	[96]
	Temozolomide	Glioblastoma	[110]
GSDMD	HER2-targeting drugs	Breast and gastroesophageal Ca	[111]
	Nelarabine, fluphenazine, dexrazoxane, bortezomib, midostaurin, and vincristine.	Renal clear cell Ca	[112]
MGLL	Progesterone	Endometrial adeno Ca	[113]
LRRFIP1	Gemcitabine	Pancreas Ca	[114]
	Teniposide	Glioblastoma	[115]
TAX1BP3	Metformin	Hepatocellular Ca	[116]
RBBP7	Cyclophosphamide, doxorubicin, and 5-fluorouracil	Basal-like breast cancer	[117]
ACTG1	Sorafenib	Hepatocellular Ca	[118]
METAP2	Fumagillin	Diverse	[119]
SUPT6H	Cisplatin	Ovarian Ca	[120]
DDX20	Epidermal growth factor	Lung Ca	[121]
MDH2	Ripretinib	Gastrointestinal stromal tumor	[122]
	Doxorubicin	Uterine cancer	[123]
	Docetaxel	Prostate Ca	[124]
RAP1GDS1	5-Fluorouracil	Colorectal Ca	[125]
TIMM23	Staurosporine	Breast Ca	[126]
	Cisplatin	Ovarian Ca	[127]
	Cisplatin	High-grade serous ovarian Ca	[128]
RPA3	Temozolomide	Glioblastoma	[129]
	Cisplatin	Lung adenocarcinoma	[130]
NCAPH	5-Fluorouracil	Colon adenocarcinoma	[131]
	Cisplatin	Oral Squamous Cell Ca	[132]
	Carboplatin	Serous ovarian cancer	[133]
FABP7	Anthracyclines and taxanes	Breast Ca	[134]
TYRP1	Cisplatin	Melanoma	[135]
	Vemurafenib and trametinib	Melanoma	[136]

## Data Availability

The original contributions presented in this study are included in the article. Further inquiries can be directed to the corresponding author.

## Abbreviations

The following abbreviations are used in this manuscript:

ABCG2	ATP-binding cassette sub-family G member 2
AMPK	AMP-activated protein kinase
GPX	Glutathione peroxidase
DAPI	4′,6-Diamidino-2-phenylindole
DDT	Dichlorodiphenyltrichloroethane
GFP	Green fluorescent protein
dlg	Docking log
FOXO1	Forkhead box O1
NCI	National Cancer Institute
Nrf2	Nuclear factor erythroid 2-related factor 2
mTOR	Mammalian target of rapamycin
NQOq	NAD(P)H quinone oxidoreductase 1
pKi	Predicted inhibition constant
pdb	Protein Data Bank
SIRT1	Sirtuin
PIK3CA	Phosphatidylinositol-4,5-bisphosphate 3-kinase catalytic subunit α
TCM	Traditional Chinese medicine
TUBA	α-tubulin
WT1	Wilms’ tumor gene
VMD	Visual Molecular Dynamics
